# Initial observations of Jinhua Qinggan Granules, a Chinese medicine, in the mitigation of hospitalization and mortality in high-risk elderly with COVID-19 infection: A retrospective study in an old age home in Hong Kong

**DOI:** 10.3389/fmed.2022.948149

**Published:** 2022-07-27

**Authors:** Timothy P. H. Lin, Edith M. C. Lau, Kelvin H. Wan, Linda Zhong, Enne Leung, Chung Nga Ko, Aiping Lu, Muhammad R. Shah, Zhaoxiang Bian, Dennis S. C. Lam

**Affiliations:** ^1^Hong Kong Alliance of Integrated Medicine Against Covid, Hong Kong, Hong Kong SAR, China; ^2^School of Chinese Medicine, Hong Kong Baptist University, Hong Kong, Hong Kong SAR, China; ^3^Center for Bioequivalence Studies and Clinical Research (CBSCR), ICCBS, University of Karachi, Karachi, Pakistan

**Keywords:** Jinhua Qinggan Granules (JHQG), Chinese medicine, SARS-CoV-2, Omicron, COVID-19

## Introduction

The coronavirus disease 2019 (COVID-19) caused by the novel severe acute respiratory syndrome coronavirus 2 (SARS-CoV-2) was first reported in December 2019 and was characterized as a pandemic on 11th March 2020 by the World Health Organization (WHO). As of 1st May 2022, it has resulted in over 500 million infections globally, with more than 6 million deaths. The advent of the highly contagious Omicron variant since November 2021 further exacerbated the disease dissemination globally. In Hong Kong, the Omicron outbreak has rapidly resulted in over 1.1 million cases among its ~7.4 million population and over 9,000 deaths, which at a time led Hong Kong to suffer as a region with the world's highest COVID-19 death rate (1). COVID-19 therefore remains a pressing grave threat to global public health.

Although various vaccines have been shown to substantially reduce hospitalization and mortality in COVID-19, a suboptimal vaccination rate, in particular among the elderly, remains one of the leading reasons for progression to severe disease and mortality. Whilst the majority of COVID-19 infections are asymptomatic and mild in nature, elderly patients and patients with various concurrent medical conditions such as cardiovascular disease, diabetes mellitus, obesity and malignancy are at high risk of progression to severe COVID-19 and its associated mortality (2, 3). It is therefore necessary to explore safe and effective anti-COVID-19 treatments which can effectively prevent the progression of COVID-19 to avert the adverse outcomes. Such therapeutic agents should best be readily available for administration to patients with mild COVID-19 at disease onset.

Oral antiviral agents currently available under emergency use authorization by the United States Food and Drug Administration for COVID-19 include Molnupiravir and Nirmatrelvir-Ritonavir. Both of these antivirals were supported by large scale randomized controlled trial to reduce the risk of progression to severe disease and mortality in patients with COVID-19 infections (4, 5). Nonetheless, it was also evident from the respective clinical trials that these antivirals may be associated with potential adverse events. In particular, as a potent antiviral nucleoside analog drug, there were concerns over the genetic safety and genotoxicity of molnupiravir (6). Traditional Chinese medicine (TCM) has often been perceived as safer and with better patient tolerability (7). Various TCMs have been approved by China's National Administration of TCM for the management of COVID-19. In the *Diagnosis and Treatment Protocol for Novel Coronavirus Pneumonia (Trial Version 7)* (8) released by China's National Health Commission & National Administration of TCM, Jinhua Qinggan Granules (JHQG) was recommended for the treatment of fatigue and fever in COVID-19 patients (8). Nevertheless, there lacks evidence in the existing literature to support the clinical efficacy of TCM in the reduction of hospitalization and mortality of COVID-19 patients, hindering clinicians from utilizing TCM in the management of COVID-19 infection. Recently, the WHO has also recommended the consideration of the potential use of TCM for the management of COVID-19, and research to further evaluate the clinical benefits and safety of TCM in the management of COVID-19 (9).

JHQG is a registered TCM in Hong Kong and mainland China and is readily available in the community. In the past, JHQG has been utilized in the treatment of the H1N1 influenza, and has been shown to effectively alleviate symptoms and promote recovery among influenza patients (10, 11). Our group was involved in a recent study that demonstrated that JHQG was effective in promoting recovery from COVID-19 related symptoms and suppressing acute phase reactants in a group of Pakistani patients with mild COVID-19 infections (12). Nonetheless, there is currently no evidence in the literature to evaluate the efficacy of JHQG in the reduction of hospitalization and mortality of COVID-19 patients who are at high risk of progression. Furthermore, the efficacy of JHQG against the novel Omicron variant, which is the currently dominant SARS-CoV-2 variant, remains undetermined.

Although the Omicron variant demonstrates hyper-transmissibility compared to earlier SARS-CoV-2 variants (13), evidence has shown that infection of Omicron was mainly limited to the upper respiratory tract due to tropism shift and attenuated host cell entry and replication competence at the lower respiratory tract (13, 14). As a result, the Omicron variant manifested reduced severity compared to earlier Delta variant infections (15, 16). Despite the reduced hospitalization and mortality reported in epidemiological studies, the Omicron outbreak in Hong Kong has resulted in overwhelming hospitalizations and many deaths among the elderly population (1). The marked adverse outcomes of Omicron outbreak in the elderly population may potentially be attributed to deconditioning, poor feeding and dehydration of frail elderly patients with poor premorbid status secondary to the development of upper respiratory tract infection (URTI) symptoms and compromised general conditions (17). As JHQG has been shown to effectively alleviate URTI symptoms in influenza and COVID-19 patients, we therefore hypothesize that administration of JHQG in the early course of Omicron infection may potentially reduce subsequent progression and adverse outcomes in elderly patients through its effective symptomatic relief.

We conducted a retrospective case review in March 2022 at an old age home with nosocomial outbreak of COVID-19 among its residents in February to March 2022, during which JHQG were distributed and administered by its residents, to evaluate the efficacy of this TCM.

## A retrospective case series at an old age home showing JHQG had the ability to prevent hospitalization and death

A nosocomial outbreak of COVID-19 occurred since 23rd February 2022 at an old age home in Hong Kong. Many of the 84 elderly residents (mean age: 81 years), all with incomplete COVID-19 vaccination status, presented with symptoms of COVID-19 infection. The diagnosis of COVID-19 was subsequently confirmed by rapid antigen test (RAT). On 28th February 2022, six patients had required hospitalization, and 4 eventually succumbed to COVID-19 infection. On 1st March 2022, JHQG were offered to the remaining 78 residents. Three declined to receive JHQG while the other 75 residents received a course of JHQG (5 g/sachet) orally 3 times per day for 10 days (30 doses in total). Six of these 75 residents (8%) did not develop COVID-19 infection while the remaining 69 residents were diagnosed of COVID-19 infection with RAT. None of the 69 patients who completed the course of JHQG progressed to severe COVID-19 infection which resulted in hospitalization or death. Among the 3 residents who did not take JHQG, all progressed to severe COVID-19 infection which required hospitalization, and two eventually succumbed to the infection (Table 1). Fisher's Exact Test showed that the use of JHQG significantly reduced the risk of progression to hospitalization and death in elderly COVID-19 patients (both *Ps* < 0.001) (Table 2).

**Table 1:** Demographics and clinical data of patients progressing to hospitalization and death.

**Case**	**Age**	**Gender**	**Past medical history**	**Outcome**
1	75	Male	History of nasopharyngeal carcinoma	Death
2	100	Male	Nil	Death
3	92	Male	Stroke, Ryle's tube feeding	Death
4	88	Male	Renal failure, diabetes mellitus, hypertension	Death
5	98	Male	Hypertension, dyslipidaemia	Death
6	80	Female	Hypertension, dyslipidaemia	Death
7	78	Female	Hypertension	Hospitalization

All patients did not take JHQG; The outbreak of COVID-19 was on 23rd February 2022 and there were 4 patients (1–4) who succumbed to the infection 5 days afterwards (28th February 2022); Three patients (5–7) declined the use of JHQG (offered on 1st March 2022), all of them required hospitalization and two of them succumbed to the infection.

**Table 2:** Efficacy of JHQG in reducing hospitalization and mortality in high risk elderly patients.

**Exposure/Outcome**	**Number of patients**	**Number of patients**	
	**Hospitalization**	**No hospitalization**	***P-*value^†^**
Took JHQG	0	69	
Did not take JHQG	9	0	<0.001
	**Death**	**Recovered**	
Took JHQG	0	69	
Did not take JHQG	6	3	<0.001

JHQG, Jinhua Qinggan Granules.

^†^Fisher's exact test.

We also obtained nasopharyngeal swabs from 66 of the 69 residents who have completed the course of JHQG and recovered from the COVID-19 infection for SARS-CoV-2 reverse transcription polymerase chain reaction (RT-PCR) to assess the viral load of these recovered patients. Three patients were on home leave at the time of site visit to the old age home for obtaining the specimens for SARS-CoV-2 RT-PCR, their viral loads were hence not available for this analysis. The average time from COVID-19 diagnosis to the time of PT-PCR testing was 25 days. SARS-CoV-2 were undetectable in 50 samples (75.8%), whilst the Ct values of 9 samples (13.6%) were ≥35 and those of 7 samples (10.6%) ranged between 30 and <35, respectively (Table 3). At the time of review, no severe adverse events (AE) potentially related to the use of JHQG have been reported. From a recent meta-analysis by Wang et al. which analyzed the clinical efficacy of Chinese herbal medicine in the treatment of COVID-19, it was also found that no previous studies reported any serious AE in COVID-19 treated with TCM (18). It is noteworthy that a previous randomized controlled trial using JHQG for the treatment of COVID-19, diarrhea was observed to be a statistically significant side effect in patients treated with JHQG, compared to the control group (19). Nonetheless, we did not observe such side effects in our current cohort.

**Table 3:** SARS-CoV-2 RT-PCR results of 66 elderly COVID-19 patients after administration of JHQG in the convalescent period.

**Result/Ct value**	**Number of samples (%)**
Undetectable	50 (75.8%)
Detectable (Ct ≥ 35)	9 (13.6%)
Detectable (Ct 30 to <35)	7 (10.6%)

Three out of 69 patients who were given and used JHQG on 1st March were on home leave on the day of specimen collection for SARS-CoV-2 RT-PCR, hence only 66 RT-PCR results from the recoverees were analyzed.

## Discussion

It is known that the risk of progression of COVID-19 and its related hospitalization and mortality increased markedly with increasing age (2, 3). In this retrospective case series, we presented preliminary evidence to that JHQG could potentially reduce hospitalization and mortality of elderly COVID-19 patients with incomplete vaccination who were at risk of progression to severe disease. Furthermore, we also showed that viral loads inferred from SARS-CoV-2 RT-PCR remained consistently low in patients who have taken JHQG in the convalescent period. To the best of our knowledge, this is the first report in the literature examining the efficacy of JHQG in reducing hospitalization and mortality among COVID-19 patients. Our findings provided new evidence to support the use of this TCM in the treatment of COVID-19 patients, in particular elderly, at risk of progression to severe disease.

JHQG is one of the “3 Medicines and 3 Formulations” recommended as TCM effective for treatment of COVID-19 patients (8), and is itself a repurposed existing TCM previously used for symptomatic relief of respiratory illnesses including SARS, H1N1 influenza and pneumonia among the 3 Medicines (JHQG, Lianhua Qingwen Capsules and Xuebijing injection) (11, 18). Various chemical components of JHQG have been identified, such as kaempferol, stigmasterol, and quercetin (20). These compounds possess anti-viral, anti-inflammatory, and immune regulatory effects (20), and have been shown in molecular docking analysis to bind to angiotensin converting enzyme II (ACE2) receptor on host cells (21), which may hence prevent viral entry and inhibit the viral activity of SARS-CoV-2 within hosts and mitigate disease progression (22). Recent network pharmacological analyses have further identified additional active pharmacological compounds in JHQG, including rutin, luteolin, wogonin, myricetin, quercetin, ursolic acid, chrysoeriol, and glabridin (23–25). These compounds are involved in complex webs of signaling and metabolic cascades associated with immune regulation, anti-inflammation, and protection from oxidative stress and tissue injury (23–25). Furthermore, immunopharmacological investigations have revealed that JHQG rapidly induced a significant decrease in plasma interleukin (IL)-6 and neutrophil/lymphocyte ratio (NLR), and a significant increase in plasma interferon gamma (IFN-γ) (25). It is known that COVID-19 could induce an aberrant surge in plasma IL-6 levels, culminating in the “cytokine storm” and systemic hyperinflammation (26). The plasma IL-6 level is also associated with the severity and mortality of COVID-19 (27). Meanwhile, IFN-γ has direct antiviral effects and is involved in viral clearance through potentiating the host immune responses (28). The therapeutic efficacy of JHQG in mitigating the progression of COVID-19 and its mortality may therefore potentially be associated with its immunomodulatory activities.

We recently completed a double-blind, placebo-controlled, randomized controlled trial conducted in non-hospitalized Pakistani patients with mild COVID-19 infection (12). The findings, under review, demonstrated that early administration of JHQG led to effective relief of viral infection symptoms. Such findings corroborate with our observations during our site visit to the old age home where recoverees of the Omicron infection reported subjective improvement in URTI symptoms, such as cough and sore throat after the use of JHQG. The effectiveness of JHQG in improving URTI symptoms such as recovery of phlegm was further evidenced by the findings of a recent meta-analysis evaluating the efficacy of TCM in the treatment of COVID-19 (18). Whilst Omicron infection is limited to the upper respiratory tract and mild in nature in the vast majority of patients as reported in epidemiological data (15, 16), it is crucial to address URTI symptoms and general conditions of viral infection in frail elderly patients who are at risk of subsequent deterioration, hospitalization and death due to physical deconditioning, dehydration and malnutrition after the development of such symptoms (29, 30). As JHQG could potentially alleviate such symptoms, it is therefore plausible that early administration of JHQG to high risk non-hospitalized patients with Omicron infection could potentially mitigate subsequent progression and adverse outcomes by preventing physical deconditioning secondary to the early symptoms of COVID-19 infection. On the other hand, findings from a meta-analysis also concluded that the use of JHQG led to improvement and recovery of chest computed tomography (CT) manifestations in COVID-19 patients (18), providing additional evidence to suggest the clinical efficacy of this TCM for patients at the more severe end of the spectrum of COVID-19 infection.

We acknowledge limitations of this preliminary study. Firstly, the sample of study was small and only included elderly patients, which limits the generalizability of our findings to patients with other co-morbidities who are also at risk of COVID-19 progression and the general population. In addition, as a retrospective study, we lacked a control group for comparison, in particular for comparison of the sequential changes in serological markers and imaging findings following the use of JHQG in COVID-19 patients which may provide important clinical information regarding the safety profile and efficacy of this TCM. Finally, confounding morbidities could also lead to bias. Future prospective studies would therefore be warranted to confirm our initial findings and the role of JHQG in the treatment of COVID-19 and potentially other respiratory infections.

In conclusion, we observed in this retrospective case series that a short course of JHQG administered at the onset of COVID-19 was safe and effective in reducing hospitalization and mortality in elderly COVID-19 patients who are at risk of progression. Moreover, the viral loads remained low after recovery in these COVID-19 patients.

## Author contributions

All authors listed have made a substantial, direct, and intellectual contribution to the work and approved it for publication.

## Conflict of interest

The authors declare that the research was conducted in the absence of any commercial or financial relationships that could be construed as a potential conflict of interest.

## Publisher's note

All claims expressed in this article are solely those of the authors and do not necessarily represent those of their affiliated organizations, or those of the publisher, the editors and the reviewers. Any product that may be evaluated in this article, or claim that may be made by its manufacturer, is not guaranteed or endorsed by the publisher.
